# Supplementary material for: Urine lactate concentration as a non-invasive screener for metabolic abnormalities: Findings in children with autism spectrum disorder and regression
Source: PLoS One. 2022 Sep 9;17(9):e0274310. doi: 10.1371/journal.pone.0274310 (PMC9462744; doi:10.1371/journal.pone.0274310)
Supplement: S1 File — (DOCX) [file pone.0274310.s001.docx]

**S1.***Supporting Information on the recruitment and research flow chart (T1 = Time point 1)*

**T1**

Child examination

- Biomarker lactate concentration in urine
- Cognitive functioning
- ASD-characteristics and severity

Parent report

- Clinical characteristics of mitochondrial dysfunction
- History of developmental regression

Participants included in the present study

*N* = 99

Participants excluded

*n* = 4

No official community diagnosis of ASD

*n* = 1

No urine sample provided

*n* = 3

Initial community-based recruitment

*N* = 103
