# Supplementary material for: Urine lactate concentration as a non-invasive screener for metabolic abnormalities: Findings in children with autism spectrum disorder and regression
Source: PLoS One. 2022 Sep 9;17(9):e0274310. doi: 10.1371/journal.pone.0274310 (PMC9462744; doi:10.1371/journal.pone.0274310)
Supplement: S2 File — (DOCX) [file pone.0274310.s002.docx]

**S2.**

*Supporting Information interview: anamnesis metabolic disorders*

ID:_______________________________________________________________________
Date:_____________________________________________________________________
Interviewer:________________________________________________________________
Respondent:_______________________________________________________________

Relationship of respondent with the child (circle):

Biological mother Stepmother Adoption mother

Biological father Stepfather Adoption father Other:_____________

| **Did your child as a baby** | **Yes** | **No** | **Remarks** |
| --- | --- | --- | --- |
| Had problems sitting independently? |  |  |  |
| Had problems with walking? |  |  |  |
| Had trouble controlling his/her head? |  |  |  |
| Had weak muscles or hypothonia? |  |  |  |

| ***Has your child ever suffered from*** | ***Yes*** | ***No*** | ***If YES, please specify (specific impairments or diagnosis, onset age, frequency of impairments, ,…)*** |
| --- | --- | --- | --- |
| Respiratory impairments |  |  |  |
| Eye impairments |  |  |  |
| Ear impairments |  |  |  |
| Fine motor impairments |  |  |  |
| Gross motor impairments |  |  |  |
| Cardiovascular disease |  |  |  |
| Kidney impairments |  |  |  |
| Gastro-Intestinal (GI) impairments |  |  |  |
| Liver impairments |  |  |  |
| Metabolic impairments |  |  |  |
| Extreme fatigue |  |  |  |
| Seizures |  |  |  |
