# Supplementary material for: Urine lactate concentration as a non-invasive screener for metabolic abnormalities: Findings in children with autism spectrum disorder and regression
Source: PLoS One. 2022 Sep 9;17(9):e0274310. doi: 10.1371/journal.pone.0274310 (PMC9462744; doi:10.1371/journal.pone.0274310)
Supplement: S3 File — (DOCX) [file pone.0274310.s003.docx]

**S3.**

*Supporting Information interview: Medical and psychiatric history of the child and family*

ID:_______________________________________________________________________
Date:_____________________________________________________________________
Interviewer:________________________________________________________________
Respondent:_______________________________________________________________

Relationship of respondent with the child (circle):

Biological mother Stepmother Adoption mother

Biological father Stepfather Adoption father Other:_____________

**Does the child or (a) family member(s) have the following condition(s)/disease(s)?
If yes, whom does it concern? Relationship with the child (paternal/maternal side)?**

| **Disorder or impairment** | **YES** | **NO** | **Remarks** |
| --- | --- | --- | --- |
| Autism Spectrum Disorder (ASD) |  |  |  |
| Fragile X syndrome |  |  |  |
| Tuberous Sclerosis |  |  |  |
| Neurofibromatosis |  |  |  |
| Rett syndrome |  |  |  |
| Childhood Disintegrative Disorder (CDD) |  |  |  |
| Prader-Willi syndrome |  |  |  |
| Angelman syndrome |  |  |  |
| Other chromosomal deficiencies, disorder(s) or syndrome(s)? |  |  |  |
| Congenital Rubella Syndrome (CRS) |  |  |  |
| Phenylketonuria (PKU) |  |  |  |
| Hydrocephalus |  |  |  |
| Cerebral Palsy |  |  |  |
| Intellectual disability |  |  |  |
| Strokes or other acquired brain damage |  |  |  |
| Seizures |  |  |  |
| ADHD/ADD |  |  |  |
| Anxiety disorder |  |  |  |
| Depressive disorder |  |  |  |
| Manic depressive disorder |  |  |  |
| Schizophrenia |  |  |  |
| Has been or is admitted to a hospital for a psychiatric illness |  |  |  |
| Birth defects (e.g., cleft lip or spina bifida) |  |  |  |
| Landau-Kleffner syndrome |  |  |  |
| Immunity system impairments |  |  |  |
| Mitochondrial disorder* |  |  |  |
| Diabetes type 1 |  |  |  |
| Cardiovascular disease |  |  |  |
| Muscle impairments |  |  |  |
| Liver impairments |  |  |  |
| Extreme fatigue |  |  |  |
| Other |  |  |  |

***If YES for mitochondrial disorder, which kind of disorder?**

| **Disorder** | **YES** | **NO** | **Family member** |
| --- | --- | --- | --- |
| **MELAS**  (Mitochondrial myopathy, Encephalopathy, Lactic acidosis, And Strokelike episodes) |  |  |  |
| **Leigh syndrome** |  |  |  |
| **NARP**  (Neuropathie, Ataxia en Retinis Pigmentosa) |  |  |  |
| **MERRF**  (Myoclonic Epilepsy associated with Ragged-Red Fibers) |  |  |  |
| **Alpers-Huttenlocher syndrome** |  |  |  |
| **Pearson syndrome** |  |  |  |
| **Kearns-Sayre syndrome** |  |  |  |
| **LHON**  (Leber hereditary optic neuropathy) |  |  |  |
| **CPEO**  (chronic progressive external ophtalmoplegia) |  |  |  |
| **Other** |  |  |  |
