# Supplementary material for: Urine lactate concentration as a non-invasive screener for metabolic abnormalities: Findings in children with autism spectrum disorder and regression
Source: PLoS One. 2022 Sep 9;17(9):e0274310. doi: 10.1371/journal.pone.0274310 (PMC9462744; doi:10.1371/journal.pone.0274310)
Supplement: S4 File — (DOCX) [file pone.0274310.s004.docx]

**S4.***Supporting Information on additional analyses lactate/creatinine ratio concentration in urine.*

1. **Differences in ASD characteristics and regression in the children belonging to the group with the lowest and highest lactate/creatinine ratio.** No significant differences were found in the ADOS-2 CSS and prevalence of children with and without regression between these ratio groups. For an overview of the results see S4 Table.

| **S4 Table. Differences in ASD characteristics and regression in the children belonging to the different lactate/creatinine ratio groups.** | | | | | |  |
| --- | --- | --- | --- | --- | --- | --- |
|  |  |  |  | **Differences  ASD-LowL/C and ASD-HighL/C** | | |
|  | **ASD-Low L/C**  **(*n* = 20)** | **ASD-Medium L/C**  **(*n* = 50)** | **ASD-High L/C**  **(*n* = 20)** | **Statistic (df)** | ***p*** | |
| **ADOS-2 Total CSS  *M* *(SD)*** | 6.75 (2.34) | 6.52 (2.17) | 5.55 (3.02) | 156.50 (40) ᵃ | .242 | |
| ***Mean rank*** | 22.68 |  | 18.32 |  |  | |
| **ADOS-2 SA CSS *M (SD)*** | 6.35 (2.54) | 6.40 (2.02) | 5.30 (2.74) | 161 (40) ᵃ | .301 | |
| ***Mean rank*** | 22.45 |  | 18.55 |  |  | |
| **ADOS-2 RRB CSS *M* *(SD)*** | 7.45 (2.52) | 7.42 (2.26) | 7.05 (2.61) | 177.50 (40) ᵃ | .547 | |
| ***Mean rank*** | 21.62 |  | 19.83 |  |  | |
| **ASD-NR *F (%)*** | 10 (50) | 33 (66) | 14 (70) | 1.667 (1) ᵇ | .197 | |
| **ASD-R≤36M *F (%)*** | 6 (30) | 13 (26) | 2 (10) | / ᶜ | .235 | |
| **ASD-R > 36M *F (%)*** | 4 (20) | 5 (10) | 4 (20) | / ᶜ | 1.000 | |
| ASD-LowL/C= children within the low lactate/creatinine ratio group, ASD-MediumL/C = children within the medium lactate/creatinine group, ASD-HighL/C = children within the high lactate/creatinine group, ADOS-2 = Autism Diagnostic Observation Scale Second Edition, CSS = Calibrated Severity Score, SA = Social Affect, ASD-R≤36M = children with regression before or at 36 months, ASD-R>36M = children with regression after 36 months, ᵃ Mann Whitney U Test, ᵇ Chi square test, ᶜ Fisher’s exact test | | | | | | |

1. **Lactate/creatinine ratio in urine between regression groups.** No significant difference was found in the lactate/creatinine ratio concentration in urine of ASD-NR (*M* = 15.9 mg/dL, *SD* = 7.6 mg/dL; mean rank = 46.9 mg/dL) and ASD-R (*M* = 15 mg/dL, *SD* = 10.7 mg/dL; mean rank = 43 mg/dL; *U*(90) = 859, *p* = .495). With regard to the 36 months age cut-off, also no significant difference was found between ASD-NR, ASD-R≤36M (*M* = 13.7 mg/dL, *SD* = 4.9 mg/dL; mean rank = 43.2 mg/dL) and ASD>36M (*M* = 17.4 mg/dL, *SD* = 16.7 mg/dL; mean rank = 42.67 mg/dL; *H*(2) = .469, *p* = .791).
